# Editorial: Electrocatalysis Towards Carbon-Neutral Future

**DOI:** 10.3389/fchem.2023.1159716

**Published:** 2023-02-20

**Authors:** Qiu Jiang, Kun Jiang, Yifan Ye, Kai Xi, Chuan Xia

**Affiliations:** ^1^ Yangtze Delta Region Institute, University of Electronic Science and Technology of China, Huzhou, China; ^2^ School of Materials and Energy, University of Electronic Science and Technology of China, Chengdu, China; ^3^ Interdisciplinary Science Research Centre, School of Mechanical Engineering, Shanghai Jiao Tong University, Shanghai, China; ^4^ National Synchrotron Radiation Laboratory, University of Science and Technology of China, Hefei, China; ^5^ School of Chemistry, Xi’an Jiaotong University, Xi’an, China

**Keywords:** electrocatalysis, environment, carbon neutrality, energy storage, energy conversion

The burning of fossil fuels has led to a significant increase in the concentration of carbon dioxide in the atmosphere, leading to global warming and other environmental impacts. The Intergovernmental Panel on Climate Change has estimated that limiting global warming to 1.5°C above pre-industrial levels will require reducing CO_2_ emissions by 45% from 2010 levels by 2030 and reaching net-zero emissions by 2050 (Masson-Delmotte et al., 2018). While the transition to renewable energy sources, such as wind and solar power, has made some progress in reducing carbon emissions, it is clear that more must be done to achieve a carbon-neutral future. Electrocatalysis has a crucial role to play in this effort, as it offers a more efficient and sustainable alternative to traditional chemical reactions. The process involves using electric potential to drive chemical reactions that reduce or oxidize substances, enabling efficient energy conversion and storage. For example, while traditional combustion-based power plants emit large amounts of carbon dioxide, fuel cells powered by hydrogen and oxygen can produce electricity with zero carbon emissions. As the world increasingly seeks ways to reduce its carbon footprint, electrocatalysis is becoming an increasingly important area of research. The articles collected in this Research Topic explore a broad range of electrocatalytic processes that are being extensively investigated, starting from basic research about the materials’ design, through to the development and validation of analytical techniques, and the evaluation of key parameters in various electrocatalytic processes.

Electrocatalytic processes can be used to directly remove carbon dioxide from industrial emissions, converting it into more manageable forms that can be safely stored. This not only reduces carbon emissions but also opens new avenues for the use of carbon dioxide as a raw material in the production of chemicals, fuels, and other products (Ding et al., 2023). Integrated Cu-based Metal-Organic Frameworks (MOFs) and their derivatives in the production of C_2+_ chemicals were summarized by Jana et al. The main advantages of using MOFs as catalysts for CO_2_ reduction are their large surface area, tunable pore size, and high stability. These properties make MOFs ideal candidates for catalyzing CO_2_ reduction reactions by providing a large active site for the reaction to occur and facilitating the transport of reactants and products. The electrocatalytic CO_2_ reduction can be potentially integrated with other processes, such as photocatalytic reaction, energy storage, or waste utilization, to improve the overall efficiency and sustainability of the process. For example, trityltetra (pentafluorophenyl) borate (TPP)-based solar cells were reported to show 23.03% power conversion efficiencies (Guo) and >90% faradaic efficiencies have already been reported for several electrocatalytic CO_2_ reductions products, combining these two processes can potentially achieve >18% energy efficiency, surpassing the theoretical efficiency of the photosynthesis process (11%) (Zhu et al., 2010). In addition to its applications in energy conversion, the electrolysis can also be potentially combined with photothermal process in certain applications to enhance their performance. Liu et al. reported that Ag-based photothermal foam with excellent light absorption and low thermal conductivity (Wang et al.), the material can efficiently evaporate water under Sun irradiations. Theoretically, the photothermal effect can be used to generate electrons and drive the electrocatalytic reaction, making it more efficient and reducing the amount of energy required. For example, photothermal electrocatalysis can be used in the reduction of CO_2_ to valuable chemicals, such as formic acid or methanol. Further mechanism-guided strategies for process integration involve the integration of CO_2_ reduction processes with other processes based on knowledge of the underlying CO_2_ reduction mechanisms (Li et al., 2022; Zheng and Xia, 2023).

Another area where electrocatalysis is making a significant impact is the hydrogen energy. One of the widely explored electrocatalytic reactions is the oxygen evolution reaction (OER). OERs occur at the anode of an electrochemical cell and involve the oxidation of water molecules to form oxygen, hydrogen ions, and electrons. OER is a critical step in many energy-related and environmental applications, such as water electrolysis, fuel cells, and water treatment. However, OER can also be challenging due to their high energy requirements and low efficiency, making it important to develop new catalysts and materials to improve the efficiency of OERs. To overcome the sluggish kinetics of OER, Zhu et al. reported an interface coupling strategy to prepare NiFe-based layered double hydroxides (NiFe-LDH) on exfoliated black phosphorous (Fan et al.). The prepared materials significantly lowered the activation energy of the OER reaction and improved its efficiency under alkaline conditions (∼240 mV overpotential at a current density of 10 mA/cm^2^). Oxygen reduction reaction (ORR) is the reverse of the oxygen evolution, it consumes the electrons and protons generated at the anode, allowing fuel cells to produce electricity with high efficiency and low emissions but suffering from sluggish reaction kinetics. Developing new catalysts with optimized structures, compositions, and morphologies can significantly improve ORR kinetics, but the precise synthesis of the desired surface is difficult. The seed-mediated synthesis is a promising method to produce well-designed nanoparticles in scale. In this method, small seed particles are first synthesized, and then they serve as nucleation centers for the growth of larger particles. The size and shape of the resulting nanoparticles can be controlled by adjusting the conditions of the synthesis, such as the concentration of reactants and reaction time. A study addressed by He et al. gave a detailed review of the seed-mediated synthesis of platinum-based electrocatalysts for ORR (Gray et al.), the method can be potentially used for the synthesis of other well-defined nanostructures for applications beyond electrocatalysis such as drug delivery, sensors, optics, and photonics, *etc.*

Overall, the development of electrocatalysis technology is a critical step toward a carbon-neutral future. As researchers continue to advance in this area, we can expect to see even greater benefits in the future. Electrocatalysis will play a crucial role in creating a more sustainable and environmentally friendly world, whether it is through the production of clean energy, the development of efficient energy conversion/storage systems, or the improvement of industrial processes. While the potential of electrocatalysis is clear, much work remains to be done to develop new and more efficient catalysts and to scale up the use of these technologies in industry and commerce.
